# Quantifying Pancreatic Fat Using a Free‐Breathing Acquisition in Adults With Obesity Undergoing Diet‐Induced Weight Loss—Results From the LION Study

**DOI:** 10.1002/nbm.70407

**Published:** 2026-09-25

**Authors:** Philipp Braun, Christoph Zoellner, Mingming Wu, Robert Graf, Jonathan Stelter, Anna Reik, Hans Hauner, Jan Stefan Kirschke, Christina Holzapfel, Daniela Junker, Dimitrios C. Karampinos

**Affiliations:** ^1^ Institute of Radiology, School of Medicine and Health, TUM Klinikum Technical University of Munich Munich Germany; ^2^ Department of Radiology Ludwig Maximilian University of Munich Munich Germany; ^3^ Institute of Neuroradiology, School of Medicine and Health, TUM Klinikum Technical University of Munich Munich Germany; ^4^ Laboratory of Magnetic Resonance Imaging Systems and Methods Ecole Polytechnique Federale de Lausanne (EPFL) Lausanne Switzerland; ^5^ Institute of Clinical Nutritional Medicine, School of Medicine and Health, TUM Klinikum Technical University of Munich Munich Germany; ^6^ Else Kröner‐Fresenius Center for Nutritional Medicine Technical University of Munich Munich Germany; ^7^ Department of Nutritional, Food and Consumer Sciences Fulda University of Applied Sciences Fulda Germany; ^8^ CIBM Center for Biomedical Imaging (CIBM) Lausanne Switzerland

**Keywords:** free‐breathing magnetic resonance imaging (MRI), pancreatic fat deposition, proton density fat fraction (PDFF), radial stack‐of‐stars

## Abstract

A free‐breathing six‐echo radial stack‐of‐stars acquisition is evaluated as an alternative to Cartesian breath‐hold scans for pancreatic fat quantification at 3T. Pancreatic fat deposition was assessed in 18 participants with obesity enrolled in a randomized controlled lifestyle intervention study (LION) using both a Cartesian breath‐hold scan and a free‐breathing high‐resolution stack‐of‐stars sequence (scan times: 16 s vs. 2 min 55 s). Pancreatic segmentations were initially generated using the VibeSegmentator and subsequently refined manually to reduce the amount of included visceral fat. The Intra‐observer reproducibility of the manual refinement was assessed after 3 months using Dice similarity score. SNR for both scan methods was quantified using a large water phantom. Mean segmented volume and mean pancreatic proton density fat fraction (PDFF) values between radial and Cartesian scans were compared. PDFF and volume changes between baseline and post‐intervention were investigated for both techniques. Segmented pancreatic volumes and PDFF measurements demonstrated excellent agreement between the radial and Cartesian sequences (Volume: *R* ^2^ = 0.97, ICC = 0.96; PDFF: *R* ^2^ = 0.98, ICC = 0.98). Compared to the Cartesian scans, the radial scans exhibited a minor positive volume bias at higher pancreatic volumes (linear regression: 0.85*x* + 9.4 cm^3^; mean volume bias: 5.8 cm^3^, 95% LoA: −7.1 to 18.7 cm^3^), alongside a proportional PDFF bias leading to slight overestimation at lower values and underestimation at higher values (linear regression: 0.88*x* + 2.1%; mean PDFF bias −0.2%, 95% LoA: −3.0 to 2.7%). Post‐intervention, both datasets captured significant decreases in pancreatic PDFF (mean loss 2.3% ± 2.1%, Cartesian *p* < 0.001, radial *p* < 0.01) and volume (mean loss 8.4 cm^3^ ± 7.7 cm^3^; both *p* < 0.001). The present study demonstrates the feasibility of a free‐breathing radial stack‐of‐stars sequence for pancreatic fat quantification at 3T and its application in adults with obesity undergoing diet‐induced weight loss.

## Graphical abstract

This work investigates a free‐breathing six‐echo radial stack‐of‐stars acquisition as an alternative to Cartesian breath‐hold scans for pancreatic fat quantification at 3T. In 18 participants with obesity enrolled in a randomized controlled lifestyle intervention (LION), segmented pancreatic volumes and PDFF demonstrated excellent inter‐sequence agreement, with both datasets capturing significant post‐intervention decreases.

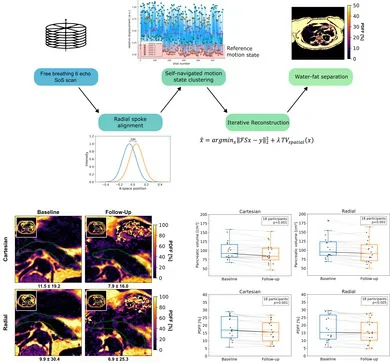

**Abbreviations**
∆TE: echo time spacing
BH: breath‐hold
CCC: Lin's concordance correlation coefficient
CI: confidence interval
CS: compressed sensing
CV: coefficient of variation
FA: flip angle
ICC: inter‐class‐correlation
LoA: limit of agreement
PCA: principal component analysis
PDFF: proton density fat fraction
SoS: stack‐of‐stars
TE: echo time
TR: repetition time

## 1 Introduction

Pancreatic fat quantification has gained increasing attention, with recent studies identifying pancreatic fat deposition as a possible risk factor for pancreatic cancer [1, 2, 3]. Fatty infiltration of the pancreatic parenchyma [4] has been linked to age [5, 6, 7, 8, 9], body mass index (BMI) [5, 6, 7] and a history of diabetes mellitus [6, 7, 8]. Moreover, a possible positive correlation between nonalcoholic fatty pancreas disease severity, mortality and systemic complications in patients with acute pancreatitis was proposed by Xie et al. [10]. However, the literature is still contradictory regarding the implications of pancreatic fat for metabolic diseases with other studies failing to verify some of the above findings [11, 12, 13, 14]. These discrepancies might be in part attributed to widely varying image acquisition and analysis methodologies, which make a consistent correlation between findings difficult especially for demanding anatomies such as the pancreas. Assessing pancreatic fat content while adhering to the current best practice for quantification may therefore provide valuable insights into metabolic and endocrine function of the pancreas.

Conventional chemical shift encoding‐based water‐fat separation techniques exploit the chemical shift difference between water and fat over multiple acquired echoes. Quantitative proton density fat fraction (PDFF) maps can then be derived from fitting a water‐fat signal model to the acquired signal evolution after consideration of multiple confounding factors [15, 16, 17]. Traditionally, acquisitions in motion‐affected areas, such as the liver or the pancreas, are acquired during breath‐holds (BHs) [18, 19, 20, 21]. However, these are constrained by the person's ability to sustain long breath‐holds, which can last up to 15–20 s while aiming for adequate high spatial resolution. Sustaining these prolonged breath‐holds is particularly challenging for pediatric, elderly, and critically ill patients. A recent work has thus investigated how the use of Compressed Sensing in combination with a deep learning reconstruction model can accelerate PDFF mapping of the pancreas under breath‐holding [22]. Yet, an incomplete breath‐hold can lead to image artifacts and spatial misregistration of the pancreatic volume [23, 24]. The pancreas is a small organ with an irregular shape and therefore requires an imaging protocol employing adequate spatial resolution and precise registration of the whole pancreatic volume. In these cases, free‐breathing acquisitions might be preferred, but these require either prospective or retrospective motion correction to mitigate motion‐related artifacts in the acquired images. Correction methods include motion signals from respiratory belts, optical sensors or navigator echoes [25, 26, 27]. Alternatively, instead of correcting for motion effects, 2D sequential acquisitions can be used to minimize motion effects by effectively freezing the motion during their short acquisition times [28, 29].

While Cartesian acquisitions can be used for free‐breathing pancreatic fat quantification at high isotropic resolutions [30], non‐Cartesian trajectories are generally more robust to motion. Radial stack‐of‐stars (SoS) trajectories have been used successfully in free‐breathing acquisitions to obtain accurate PDFF measurements even in anatomical regions such as the liver or the pancreas that are heavily affected by respiratory motion [27, 31, 32]. Specifically, Story et al. have explicitly shown that reliable quantification of the pancreatic PDFF in children is possible employing a free‐breathing radial SoS acquisition [32]. Due to the acquisition of the k‐space center in every acquisition shot, principal component analysis (PCA) can be applied across the coil elements to extract a continuous retrospective motion surrogate. This self‐navigation curve is subsequently used to group the data into different repeating motion states. This eliminates the need for any additional navigator echoes which would prolong the scan time or the need for any additional hardware to estimate the occurring motion during the sequence acquisition.

The present work aims to evaluate correlation between a high‐resolution free‐breathing radial SoS sequence and a conventional Cartesian breath‐hold acquisition for PDFF quantification in adult participants with obesity participating in a randomized controlled lifestyle intervention (LION) study.

## 2 Methods

### 2.1 Study Design and Participants

A total of 127 individuals with obesity were recruited between October 2019 and October 2021 for a randomized controlled LION study [33]. The study protocol was approved by the ethical committee of the School of Medicine and Health of the Technical University of Munich, Germany (project no. 69/19S; ClinicalTrials.gov registration no. NCT04023942). Written informed consent was obtained from all study participants. The intervention consisted of an 8‐week, formula‐based low‐caloric diet (LCD, 800 kcal/day) with an optional additional daily intake of 200 g of nonstarchy vegetables. Eighty‐one participants received a follow‐up MRI scan after completing the diet. In total, 19 participants received both the Cartesian breath‐hold scan and the proposed free‐breathing radial SoS scan (mean age 49 years, 11 female, mean BMI 34.29 kg/m^2^). One participant was excluded from further analysis due to incomplete pancreatic coverage in the radial acquisition.

### 2.2 MRI Measurements

Data were acquired on a 3T Ingenia Elition X Philips Healthcare system. The scan parameters are listed in Table 1. A clinical T2w TSE sequence was initially performed to aid in identifying the borders of the pancreas for subsequent mDixon slab placement. 3D six‐echo Cartesian BH scans were performed using Compressed Sensing (CS factor *R* = 6) during a 16.9 s breath‐hold (2 × 2 × 3 mm^3^, flip angle 3°, TR/TE _1_ / ∆TE = 8.6/1.19/1.2 ms). 3D six‐echo radial scans were performed using parallel imaging (SENSE factor *R* = 2 in the slice dimension) during a scan time of 2 min 55 s and had a higher in‐plane resolution to limit partial volume effects (1.5 × 1.5 × 3 mm^3^, flip angle 3°, TR/TE_1_/∆TE = 8.6/1.4/1.0 ms). Radial scans used a pseudo‐golden‐angle ordering [34] where one radial view was obtained along all slice encoding partitions before moving to the next radial view. A low flip angle of 3° was chosen to limit T1 bias in fat quantification.

**TABLE 1:** Imaging parameters for the Cartesian and the radial SoS pulse sequences used for fat quantification.

Trajectory	Cartesian	Radial
FOV (mm^3^)	300 × 400 × 120	450 × 450 × 120
Voxel size (mm^3^)	2 × 2 × 3	1.5 × 1.5 × 3
Number of axial slices	40	40
Water‐fat shift (pixel)	0.42	0.30
Radial spokes	N/A	601
Echoes	6	6
TE1/dTE (ms)	1.19/1.2	1.4/1.0
TR (ms)	8.6	8.6
FA α (°)	3	3
Respiratory compensation	Breath‐hold	Free‐breathing, hard‐gating
Acceleration	CS‐SENSE *R* = 6	SENSE *R* = 2
Total scan time	16.9 s	2 min 55 s

*Note:* CS‐SENSE employs a global acceleration factor using a variable‐density pseudo‐random sampling pattern jointly across both the phase‐encoding and slice‐encoding directions. SENSE in the radial SoS acquisition is applied in the slice dimension.

Abbreviations: CS: Compressed Sensing (Philips Healthcare), dTE: echo time spacing, FA: flip angle, TE: echo time.

### 2.3 Reconstruction Pipeline

PDFF maps from the Cartesian scans were obtained using the vendor's online complex‐based fat quantification algorithm (Philips mDixon quant) [35], which takes into account the complexity of the fat spectrum, single T2* decay and phase error effects.

**FIGURE 1:**
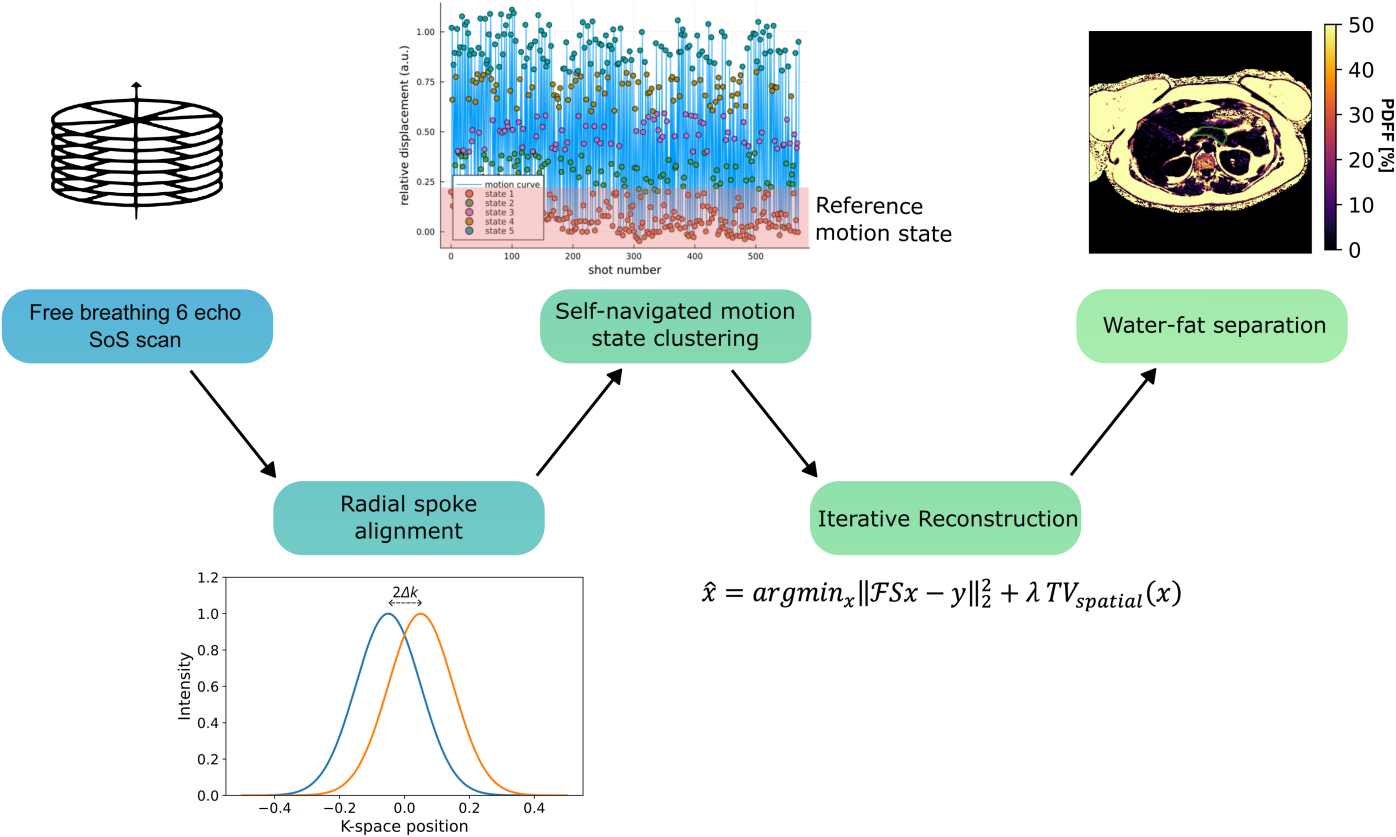
Reconstruction pipeline for a free‐breathing six‐echo radial SoS scan. Eddy currents are corrected by aligning opposite radial spokes, which are shifted towards each other by a k‐space shift 2Δ*k*. Data are binned into five different motion states using PCA‐based self‐navigation. The iterative reconstruction reconstructs only the first motion state employing a spatial total variation regularization. Water‐fat separation was performed with a hierarchical multi‐resolution graph‐cut algorithm, employing a multi‐peak fat model with a single T2* decay.

The radial PDFF maps were generated using the radial SoS trajectory. The reconstruction workflow is illustrated in Figure 1. Radial spokes were aligned by computing the linear phase offset between two opposite spokes that minimized the residual of the subtracted coil‐combined magnitude k‐space profiles to compensate for eddy current effects [36]. A PCA of the oversampled projection magnitude 1D profiles was performed to self‐navigate the data into five different motion states [37]. The following iterative reconstruction, implemented in Julia, solves the inverse problem for a chosen motion state (end‐expiration):

(1) $$\hat{x}={\text{argmin}}_{x}{\left\|F\mathrm{Sx}-y\right\|}_{2}^{2}+\lambda \;{\mathrm{TV}}_{\text{spatial}}\left(x\right)$$

where F is the nonuniform Fourier transform, and *S* represents the coil sensitivity maps derived from a pre‐scan. A spatial total variation term ${\mathrm{TV}}_{\text{spatial}}$ was included for regularization. The ADMM solver was used for the minimization problem in Equation (1). Water‐fat separation was performed with a hierarchical multi‐resolution graph‐cut algorithm, employing a multi‐peak fat model with a single T2* decay [16, 17, 38]. The Berglund 10‐peak fat model was used to model the spectral complexity of the fat peaks at 3T [39].

### 2.4 SNR Measurements

SNR was calculated according to the NEMA proposed two‐image subtraction method [40, 41, 42]. Cartesian and radial scans of a large water phantom were acquired according to the parameters detailed in Table 1. Each acquisition was performed twice, separated by an approximate 5‐min delay. To simulate the undersampling inherent to self‐navigated free‐breathing acquisitions, motion states calculated from a single volunteer were applied, and only the first motion state was reconstructed.

SNR was calculated within an automated Python‐based segmentation encompassing roughly 75% of the total phantom volume. The signal was defined as the mean of the added images while noise was determined by calculating the standard deviation (SD) of the subtracted images within the same segmentation. The SNR was then calculated as follows:

(2) $$\mathrm{SNR}=\sqrt{2}\;\frac{S_{\text{mean}}}{{\mathrm{SD}}_{\text{diff}}}$$

with $S_{\text{mean}}$ being the average signal and ${\mathrm{SD}}_{\text{diff}}$ being the SD of the subtracted images.

### 2.5 Image Analysis

**FIGURE 2:**
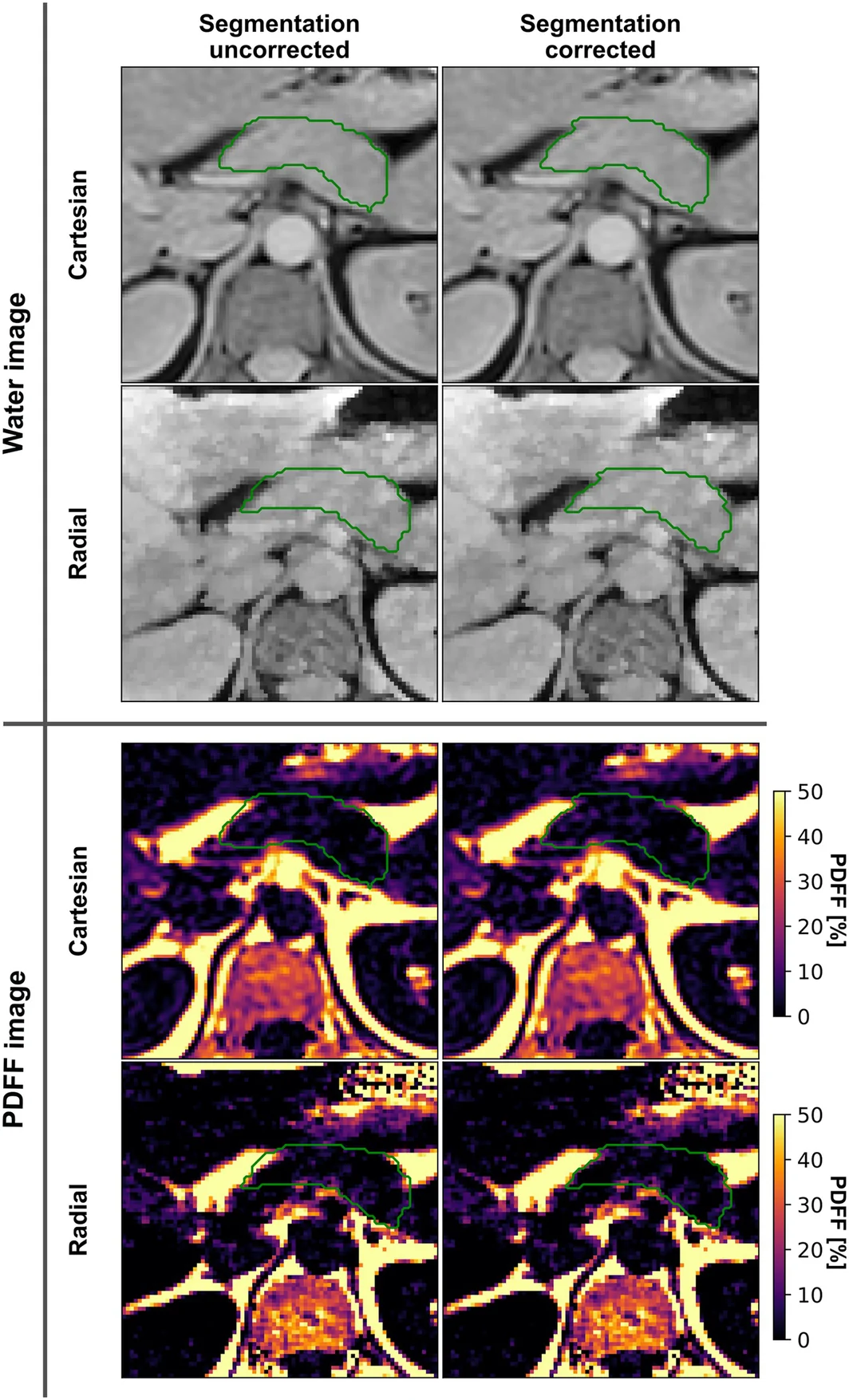
Exemplary pancreatic segmentations for one study participant before and after manual correction. The segmentation contour is shown in green. The initial segmentations were generated separately for the Cartesian and radial water images using the VibeSegmentator, which is a whole body MRI segmentation neural network. Scan times were 16 s for the breath‐hold Cartesian and 2 min 55 s for the free‐breathing radial scan. The segmentations were then manually corrected to reduce the impact of visceral fat on the pancreatic PDFF estimation. The correction was performed via overlaying the segmentation on the PDFF image. Mean pancreatic PDFF inside segmentation for Cartesian: uncorrected 6.0% ± 13.6%, corrected 5.7% ± 13.0%; Radial: uncorrected 6.1% ± 20.3%, corrected 5.6% ± 19.5%. Mean segmented pancreatic volume for Cartesian: uncorrected 85.5 cm^3^, corrected 85.1 cm^3^; Radial: uncorrected 83.8 cm^3^, corrected 83.2 cm^3^.

Pancreatic segmentations were generated separately for the Cartesian and radial water image using the VibeSegmentator, a whole body MRI segmentation neural network [43]. The generated pancreatic segmentations were then manually refined separately for both techniques to reduce the impact of visceral fat on the pancreatic fat quantification. This was done by overlaying the segmentation on top of the PDFF image in ITK‐SNAP [44]. An example of one pancreatic segmentation before and after manual correction is shown in Figure 2. The T2‐weighted TSE sequence was used for a final visual quality control of the obtained manually refined segmentations due to its superior contrast between the pancreas and surrounding tissue.

### 2.6 Statistics

Statistical calculations were performed using Python 3.8 (Python Software Foundation, https://www.python.org/). Intra‐observer reproducibility was assessed by repeating the manual segmentations after a three‐month interval and calculating the Dice similarity coefficient against the original baseline. Linearity between pancreatic PDFF from the radial and Cartesian scans was assessed by linear regression and Pearson correlation (*R* ^2^). Lin's concordance correlation coefficient (CCC) was calculated to evaluate systematic bias. The regression slope and y‐intercept were calculated alongside their respective 95% confidence intervals (CI) using the standard errors of the estimates and critical values from the two‐tailed *t*‐distribution. Mean bias, the 95% limit of agreement (LoA) and inter‐technique error coefficient of variation (CV) were assessed using Bland–Altman plots. Longitudinal PDFF changes between baseline and follow‐up visits were analyzed for normality using the Shapiro–Wilk test and a two‐sided Wilcoxon signed‐rank test with a significance level of *p* = 0.05. Data were expressed either using mean ± SD or mean (95% CI). Inter‐class‐correlation (ICC) for inter‐rater reproducibility was calculated using a single measurement, two‐way random effects with absolute agreement to check for systematic errors between the two applied measurement techniques.

## 3 Results

Manual segmentations have shown excellent intra‐observer reproducibility after repeating the manual refinement of the initial segmentations. For the Cartesian acquisition, the repeated manual segmentations demonstrated high spatial alignment with the baseline masks, yielding a mean Dice score of 0.99 (95% CI: 0.99–0.99) across the cohort. Similarly, the Radial acquisition demonstrated high reproducibility with a mean Dice score of 0.98 (95% CI: 0.98–0.99). A comparison between a T2‐weighted TSE sequence and a PDFF image of a Cartesian BH scan with the overlaid manually refined segmentation is shown in Figure S1.

**FIGURE 3:**
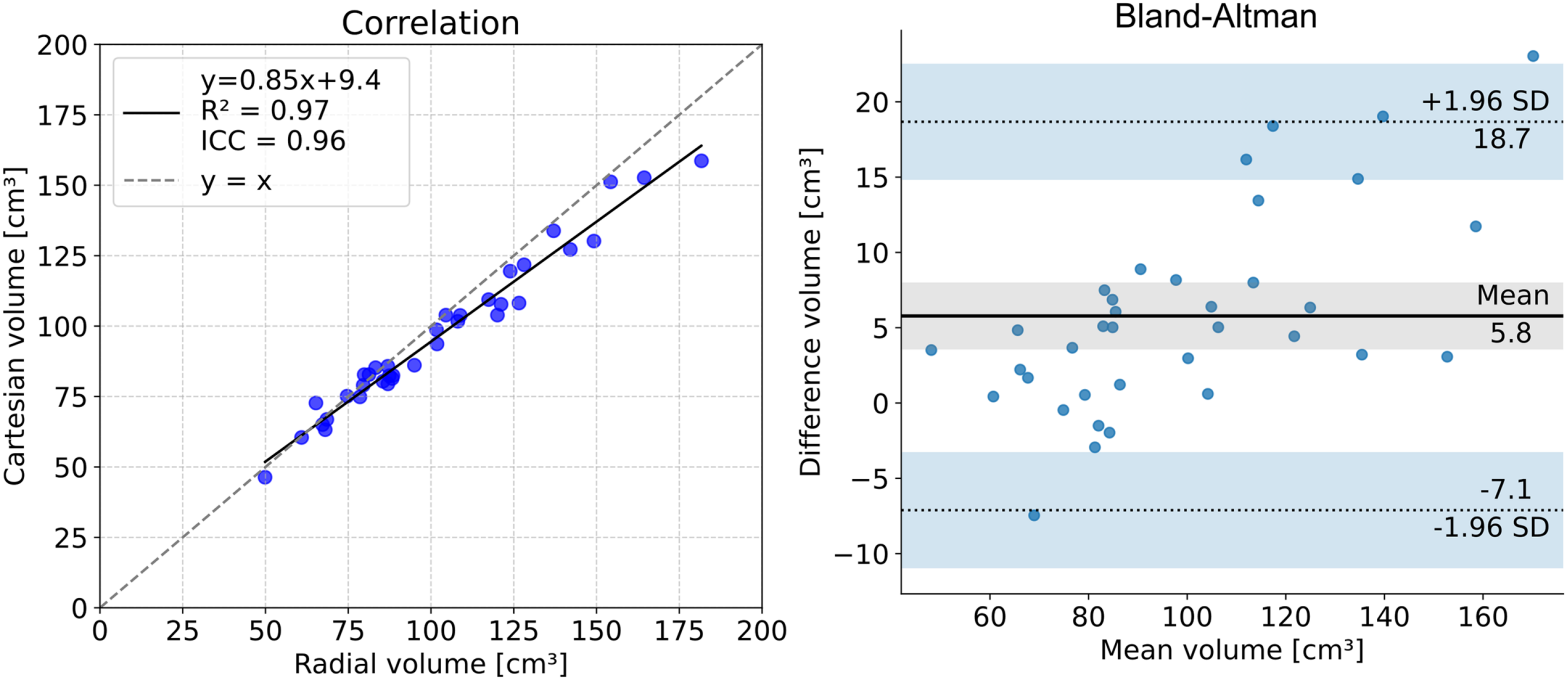
Linear regression and Bland–Altman plot for pancreatic volume segmentations obtained from the radial and the Cartesian scans for both the baseline and follow‐up visit. The segmentation procedure is shown in Figure 2. Both segmentations from the Cartesian and radial scan respectively exhibited high Pearson correlation *R* ^2^ = 0.97, inter‐rater reproducibility ICC = 0.96 (95% CI: 0.77 to 0.99), and strong overall concordance (Lin's CCC = 0.96). Linear regression yielded a slope of 0.85 (95% CI: 0.80 to 0.90) and an intercept of 9.4 cm^3^ (95% CI: 3.9–14.8 cm^3^). The Bland–Altman plot was calculated from the difference of the segmentation volumes from the radial minus the Cartesian scan. Especially for higher pancreatic segmentation volumes, the radial sequence generally shows larger segmentation volumes. Bland–Altman analysis demonstrated a mean bias of 5.8 cm^3^ with the 95% limit of agreement ranging from −7.1 to 18.7 cm^3^, corresponding to a low inter‐technique error CV of 6.6%.

A strong correlation was observed between the pancreatic volumes segmented from the radial acquisition compared with the Cartesian acquisition (Figure 3). The squared Pearson coefficient (*R* ^2^) was calculated to be 0.97 while ICC = 0.96 (95% CI: 0.77–0.99) and Lin's CCC = 0.96, indicating a strong linear correlation with minimal systematic bias between the two datasets. The linear regression analysis yielded a slope of 0.85 (95% CI: 0.80–0.90) and an intercept of 9.4 cm^3^ (95% CI: 3.9–14.8 cm^3^) showing a bias of the radial acquisition in particular towards higher segmentation volumes where the segmented volumes were overestimated compared to the Cartesian acquisition. The Bland–Altman plot shows a slight bias of 5.8 cm^3^ with 95% limit of agreement from −7.1 to 18.7 cm^3^ throughout the whole dataset, corresponding to a low inter‐technique error CV of 6.6%.

**FIGURE 4:**
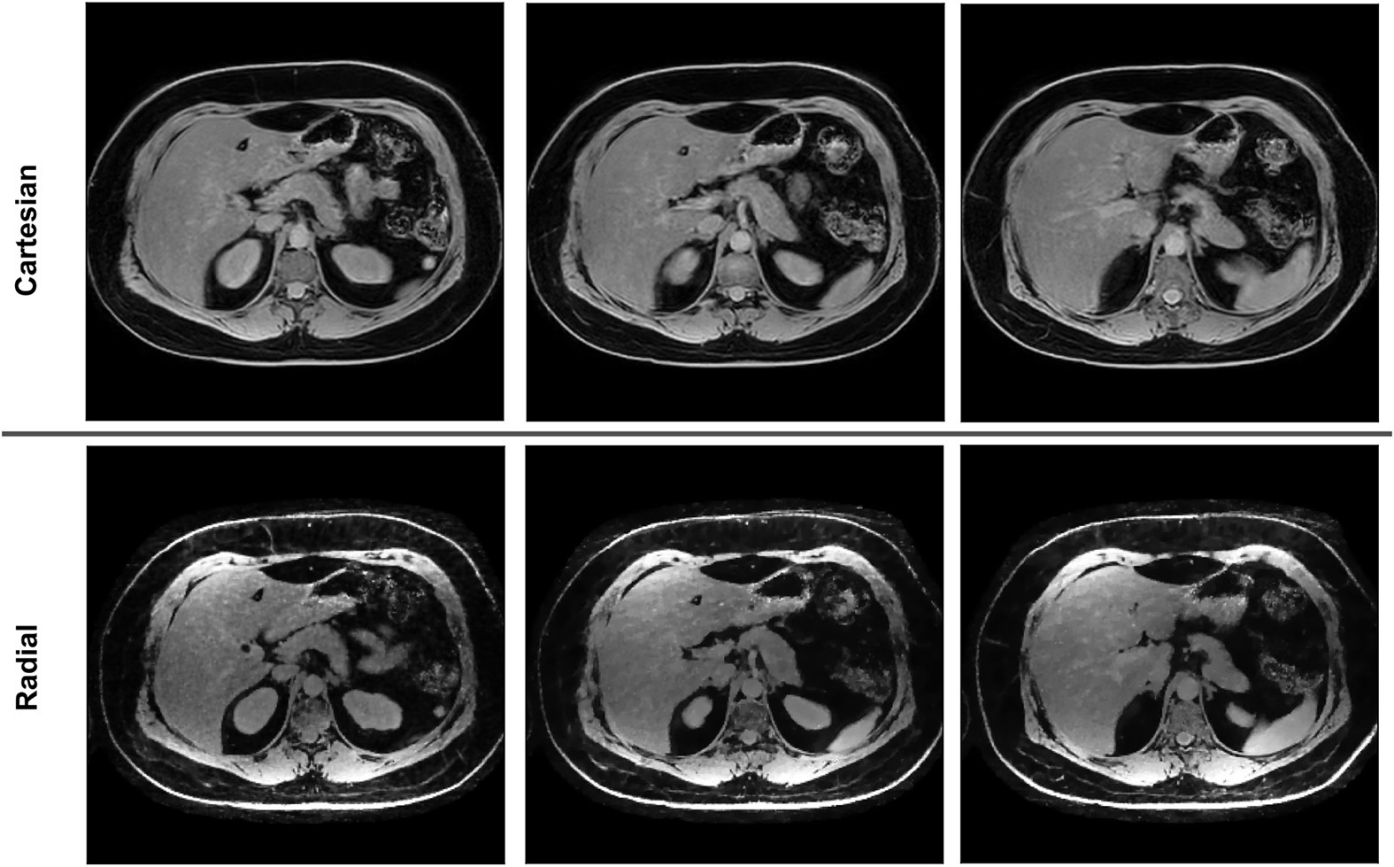
Water images of the radial and the Cartesian scan from one study participant. Scan times were 16 s for the breath‐hold Cartesian and 2 min 55 s for the free‐breathing radial scan. Different columns show different axial slice positions. Radial self‐navigation successfully removed most respiratory motion artifacts, as highlighted by the sharp delineation of the kidneys. These images highlight the higher resolution and superior delineation of the organs for the radial compared to the Cartesian sequence (Cartesian: 2 × 2 × 3 mm^3^; radial: 1.5 × 1.5 × 3 mm^3^) when motion is adequately addressed. The Cartesian sequence appears blurrier while the radial sequence appears sharper albeit showing small streaking and noise‐like artifacts. The surface coil signal intensity variation (bright signal at the anterior and posterior edges) is visible in the offline radial reconstruction due to the missing online image uniformity correction. Mean pancreatic PDFF inside segmentation for Cartesian: 10.6% ± 16.5%; Radial: 14.0% ± 27.3%. Mean segmented pancreatic volume for Cartesian: 119.5 cm^3^; Radial: 123.9 cm^3^.

**FIGURE 5:**
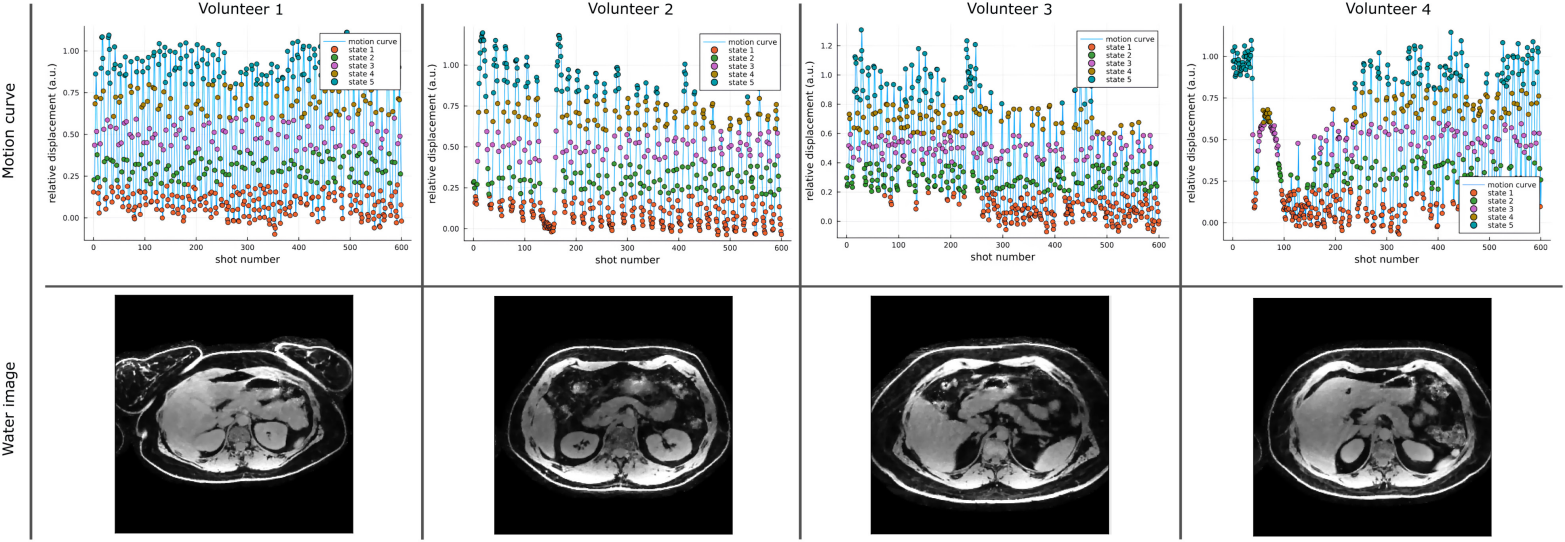
Motion curves from four volunteers derived via PCA of the oversampled projection magnitude 1D profiles, alongside a representative water image of a pancreatic cross section. For all displayed motion curves, the reconstruction pipeline successfully reconstructed high‐quality water images free of severe motion or undersampling artifacts. Notably, robust image quality was maintained for the latter two volunteers, whose motion curves were compromised by irregular breathing patterns or bulk motion during the scan.

Depending on the underlying motion curve, motion self‐navigation for the free‐breathing radial scan retained approximately 36% of the total data for reconstruction. Measurements in a large water phantom yielded high SNR for both the Cartesian (SNR = 88), fully sampled radial (SNR = 79), and motion‐navigated radial (SNR = 63) scans, though the latter exhibited a 28% decrease in SNR compared to the Cartesian scan (Figure S2). Furthermore, the motion‐navigated radial acquisition displayed mild streaking artifacts in the in‐phase images. The water images of one volunteer of multiple axial slices during a baseline scan for both the radial and the Cartesian acquisition are shown in Figure 4. Owing to the successful motion self‐navigation during reconstruction for the free‐breathing radial scan, organs such as the kidneys and liver—which would otherwise be heavily blurred by respiratory motion—appear sharp and artifact‐free. The higher resolution (1.5 × 1.5 × 3 mm^3^) of the radial sequence is apparent and a clearer delineation of the organ boundaries is visible. However, small streaking artifacts are visible inside the radial image which also appears noisier. Figure 5 displays the motion curves derived from the free‐breathing radial acquisitions of four volunteers, alongside corresponding water images of a pancreatic cross‐section. Regardless of the underlying motion profile, the reconstructed water images demonstrate high visual clarity without severe streaking or undersampling artifacts.

**FIGURE 6:**
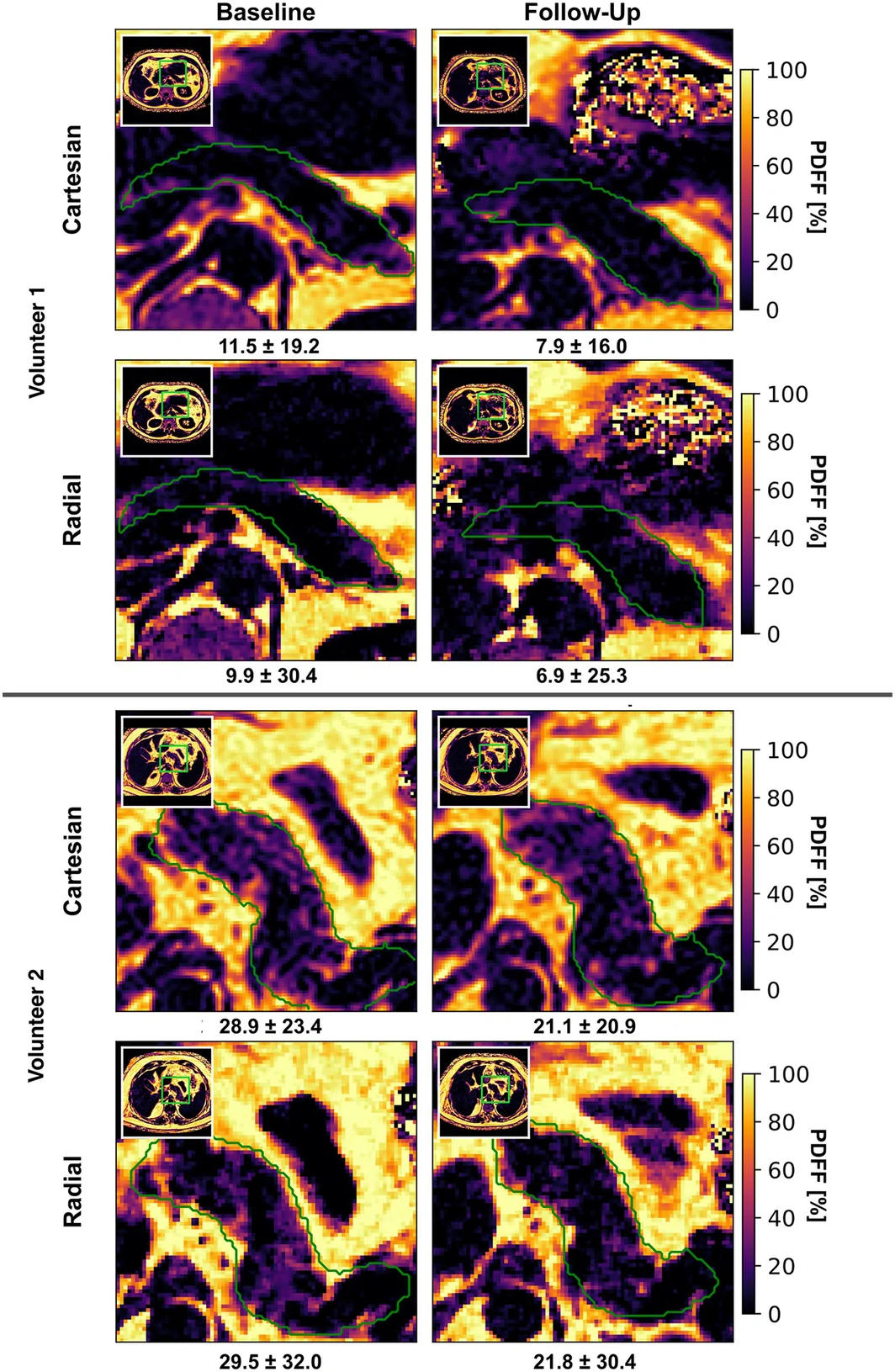
Pancreatic PDFF maps of two study participants during the baseline and 8 week later follow‐up scan. The first row shows the results from the radial scan; the second row shows the results from the Cartesian scan respectively. The whole abdominal region is shown on the top left with the pancreas region being marked with a green rectangle. The green contour highlights the segmented pancreas area. Below each image, the mean and standard deviation of the pancreatic PDFF across the entire pancreatic volume are shown. Small deviations of the anatomy can be observed due to involuntary movements during the scan session and possible deviations between the end‐expiration state from the self‐navigation and the BH state used for the Cartesian acquisition. Only slight variations of the contoured segmented pancreatic volumes can be observed between the radial and the Cartesian scan. For both scans pancreatic PDFF is reduced on the follow‐up compared to the baseline scan. Based on the Cartesian scan the pancreatic volume of volunteer 1 changed from 127.2 to 130.2 cm^3^ and of volunteer 2 from 158.7 to 152.7 cm^3^ between the baseline and follow‐up scan respectively.

Figure 6 shows representative PDFF maps from two participants at baseline and at the 8‐week later follow‐up scan. Only slight variations of the contoured segmented pancreatic volumes were observed between the radial and the Cartesian scan. The follow‐up scan shows a reduced pancreatic PDFF compared to the baseline scan.

**FIGURE 7:**
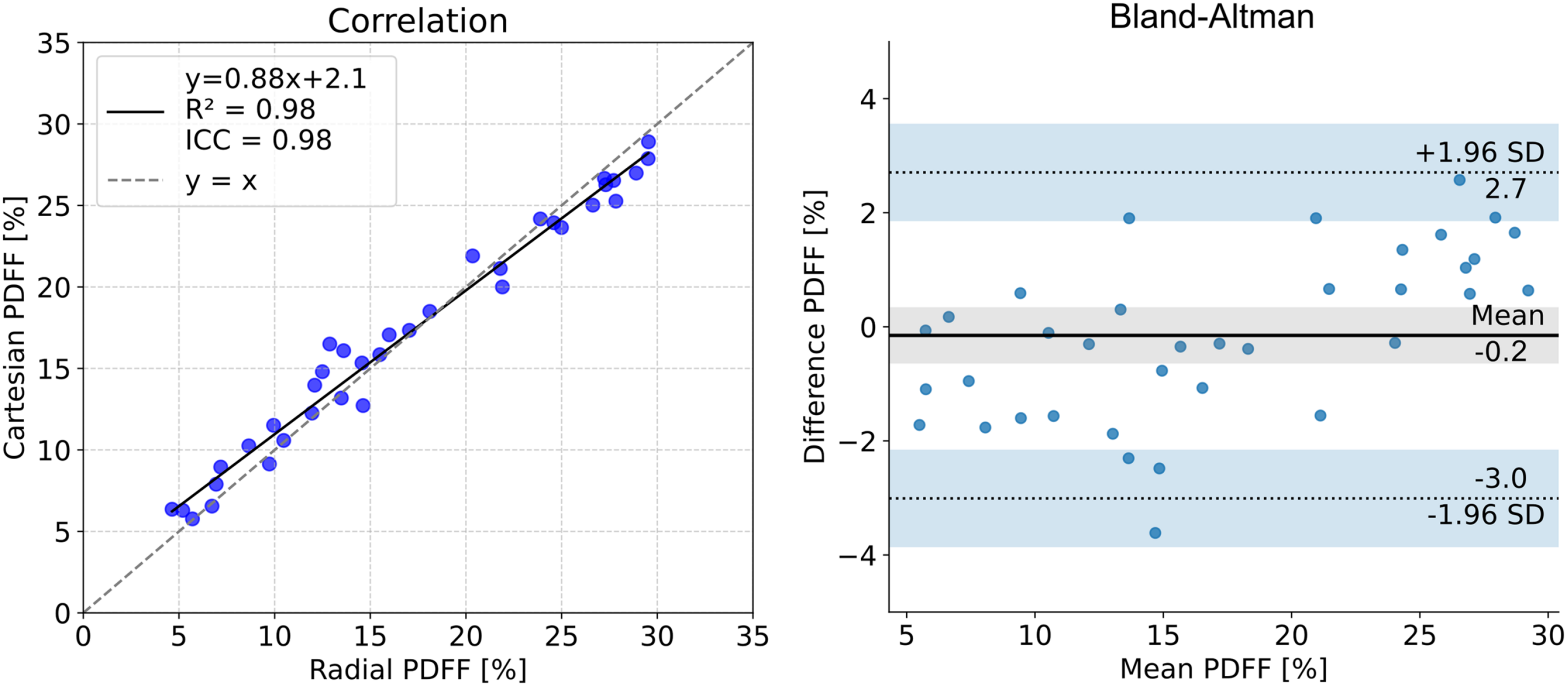
Linear regression and Bland–Altman plot of the mean pancreatic PDFF comparing PDFF data acquired from the radial scan with data acquired from the Cartesian scan for both the baseline and follow‐up visit. Both datasets from the Cartesian and radial scan respectively exhibited high Pearson correlation *R* ^2^ = 0.98, inter‐rater reproducibility ICC = 0.98 (95% CI: 0.97 to 0.99), and strong overall concordance (Lin's CCC = 0.96). Linear regression yielded a slope of 0.88 (95% CI: 0.83 to 0.93) and an intercept of 2.1 (95% CI: 1.2 to 3.0). At lower PDFF values, the radial acquisition tended to slightly underestimate pancreatic fat fraction, whereas at higher PDFF values it slightly overestimated compared to the Cartesian reference. The Bland–Altman plot was calculated from the mean pancreatic PDFF of the radial minus the Cartesian scan. Bland–Altman analysis demonstrated a mean bias of −0.2% with the 95% limit of agreement ranging from −3.0% to 2.7% PDFF, corresponding to a low inter‐technique error CV of 8.4%.

Comparison of the mean PDFF inside the segmented pancreas volume between the two scan sequences again revealed a strong linear correlation (Figure 7). The squared Pearson correlation coefficient *R* ^2^ is given by 0.98 while also expressing a high ICC = 0.98 (95% CI: 0.97 to 0.99) and a high Lin's CCC of 0.96. Linear regression yielded a slope of 0.88 (95% CI: 0.83–0.93) and an intercept of 2.1 (95% CI: 1.2–3.0) indicating a small systematic bias in mean pancreatic PDFF values calculated from the radial acquisition, compared to the Cartesian acquisition. Higher PDFF values appeared to be slightly overestimated with the radial acquisition, while lower PDFF values seem to be slightly underestimated. This can also be seen in the Bland–Altman plot, where a small mean bias of −0.2% with the 95% limit of agreement ranging from −3.0% to 2.7% PDFF is demonstrated. This corresponds to a low inter‐technique error CV of 8.4%.

**FIGURE 8:**
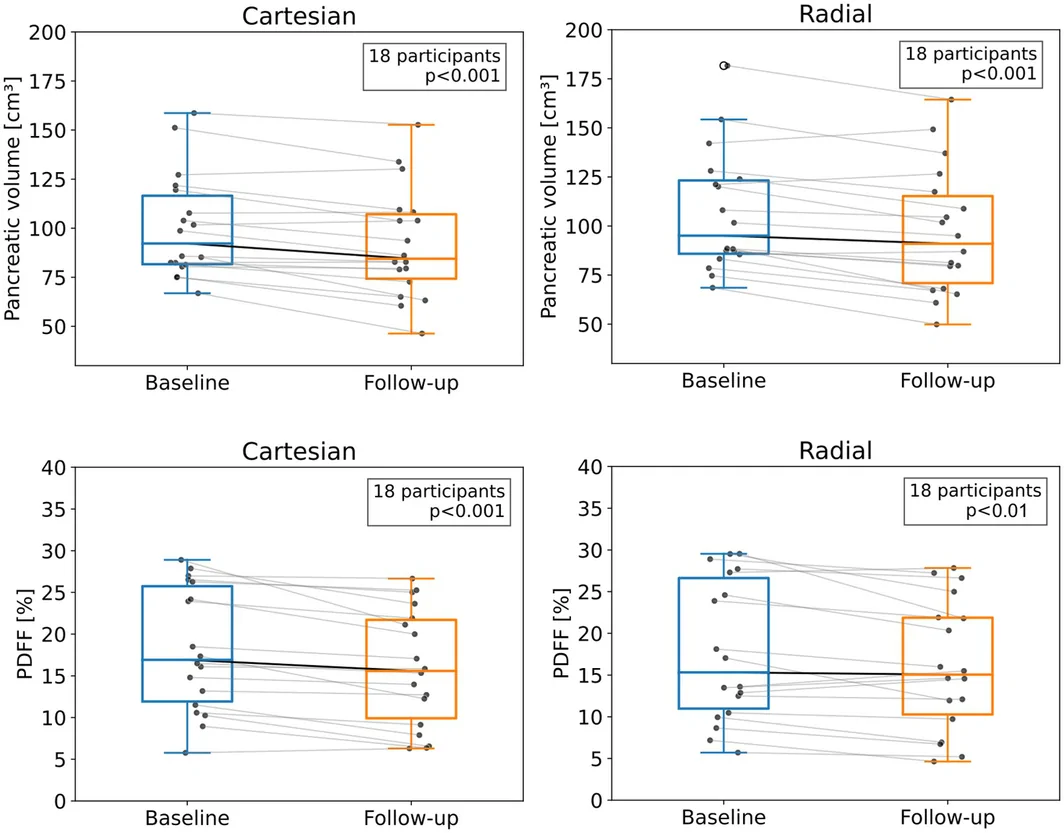
Boxplots illustrating the change in mean pancreatic PDFF and pancreatic volume between the baseline and follow‐up scan. The analysis was performed separately for both the radial and Cartesian acquisition separately. Paired‐samples t‐tests values were used to assess statistical significance. Both acquisition types demonstrated a highly significant reduction in mean pancreatic PDFF and pancreatic volume in the follow‐up scan. Mean weight loss 10.8 kg ± 2.1 kg. Based on the Cartesian scan the mean pancreatic PDFF loss was 2.3% ± 2.1% (Radial *p* <  0.01, Cartesian < 0.001) and the mean pancreatic volume loss was 8.4 cm^3^ ± 7.7 cm^3^ (Radial *p* < 0.001, Cartesian < 0.001).

Figure 8 depicts longitudinal pancreatic mean PDFF and pancreatic volume changes between the baseline and follow‐up scan for the 18 participants of the LION study. A Shapiro–Wilk test yielded p_vol_, _cart_ = 0.45, p_vol_, _rad_ = 0.34 for the Cartesian and radial volume changes and p_PDFF_, _cart_ = 0.09, p_PDFF_, _rad_ = 0.67 for the Cartesian and radial PDFF changes across the two spaced visits. While all *p*‐values were higher than the significance level of *p* = 0.05 and hence the data can be assumed to be normally distributed, a two‐sided Wilcoxon signed‐rank test was performed due to the low sample size.

This test was performed separately for both scan sequences individually. For both acquisition trajectories a significant decrease in pancreatic mean PDFF (Radial *p* < 0.01, Cartesian *p* < 0.001) and pancreatic volume (Radial *p* < 0.001, Cartesian *p* < 0.001) was observed. The mean weight loss of the participants was 10.8 kg ± 2.1 kg. Based on the Cartesian scan, the mean pancreatic PDFF decrease was 2.3% ± 2.1% and the mean pancreatic volume loss was 8.4 cm^3^ ± 7.7 cm^3^.

## 4 Discussion

This work demonstrates the feasibility of a radial SoS trajectory for free‐breathing high‐resolution fat quantification of the pancreas at 3T. The study compares mean pancreatic PDFF values from the proposed free‐breathing radial acquisition with the values from a Cartesian acquisition under a breath‐hold. The work also combines the free‐breathing acquisition with an image analysis pipeline to investigate the ability of the free‐breathing method to track mean pancreatic PDFF value changes in people with obesity undergoing a weight loss intervention.

Pancreatic fat quantification has garnered increasing attention, with recent studies linking it to a multitude of metabolic diseases [1, 2, 3, 6, 7, 8]. To mitigate motion artifacts caused by the organ's close proximity to the diaphragm, most previous studies have employed Cartesian BH scans which last between 12 and 19 s. Due to the highly irregular shape of the pancreas and the spatial heterogeneity of its fat deposition, these studies typically require higher spatial resolutions of 1.5–2.2 mm in‐plane and 3–5 mm slice thickness [13, 32, 45, 46]. However, pediatric, elderly, and critically ill patients often struggle to sustain these prolonged breath‐holds. A free‐breathing MRI sequence would make this clinical analysis accessible to a broader demographic while significantly enhancing patient comfort during scanning. Free‐breathing fat quantification techniques in the liver have already been demonstrated successfully in a pediatric patient group [47, 48]. Recent work by Story et al. demonstrated that a free‐breathing radial SoS scan can be successfully employed in a pediatric cohort while maintaining the high resolution necessary for accurate pancreatic PDFF mapping (1.7–1.9 mm in‐plane, 5 mm slice thickness) [32]. The present study achieves a higher resolution of 1.5 mm in plane and a thinner slice thickness of 3.0 mm.

While this spatial resolution is critical to minimize partial volume effects at the organ's boundaries, the inherent anatomical complexity of the pancreas still makes the accurate annotation of measured PDFF maps challenging. As reported by Kato et al. [49] and later verified by Story et al. [32], the use of ROIs in the pancreas to quantify and evaluate different PDFF measurement methods, as it is frequently done for hepatic PDFF estimation, is difficult due to the patchy nature of pancreatic fat accumulation [50]. Hence, full pancreatic volume segmentations were initially generated with the VibeSegmentator [43] to provide a standardized method for obtaining the segmentations, which is more reliable than manually setting up ROIs in the pancreas volume [22]. However, due to the difficulty of segmenting such an irregularly shaped and motion‐affected area, the Dice score of the pancreas segmentation of the VibeSegmentator is comparably low at 0.81 [43]. This led to the inclusion of some visceral fat in each segmentation, which substantially affected the subsequent PDFF estimation. To reduce this effect, each segmentation was presently manually corrected to reduce the amount of visceral fat in the segmentation. Although manual refinement introduces a potential source of variability to the analysis, intra‐observer reproducibility between segmentations performed 3 months apart was exceptionally high. The resulting 3D segmentation volumes were comparable between the Cartesian and radial scan, but the radial scan generally yielded a larger segmentation volume. This effect scales with the pancreatic volume of the study participant (Figure 3). While the higher resolution of the radial scan reduces segmentation errors caused by partial volume effects, the blurring introduced by residual motion and potential streaking artifacts following PCA‐based motion self‐navigation ultimately results in greater segmentation volumes when compared to the Cartesian scan. The SNR difference between the Cartesian and radial scan can likely be attributed to differing acquired voxel sizes (2 × 2 × 3 mm^3^ vs. 1.5 × 1.5 × 3 mm^3^), acceleration factors, and employed reconstruction techniques. Specifically, the CS‐SENSE acceleration technique used for the Cartesian acquisition applies wavelet filtering to the image, which actively denoises the image and yields higher SNR values.

There is sparse information in the literature regarding free‐breathing pancreatic fat quantification. Story et al. have characterized pancreatic PDFF spatial heterogeneity in 34 children and showed that using a free‐breathing radial SoS MRI sequence yields similar results to a clinical BH scan [32]. However, due to age‐related differences in the pancreatic volume and morphology, verification is also required for different patient cohorts [5, 6, 7, 8, 9]. In both pediatric and adult populations, irregular breathing patterns and bulk motion can impact the motion curves derived from free‐breathing acquisitions. Careful selection of the motion state and window height is required to ensure sufficient k‐space coverage and prevent radial streaking and undersampling artifacts. For all 18 adult participants in the LION cohort, sufficient k‐space sampling was successfully achieved, and no severe motion or undersampling artifacts were observed in the resulting water images (Figure 5). Comparable PDFF image quality and quantitative results within the full segmented pancreas volume were achieved. Due to the free‐breathing radial sequence not being time‐constrained, a higher resolution scan can be obtained, as apparent in Figures 4 and 6.

Small variations in the exact locations of the scanned anatomy can be observed between the radial and Cartesian scan, which can be attributed to small movements of the scanned subject during the scan protocol as well as possible anatomical differences between the breath‐hold expiration state and the estimated expiration state from the self‐navigated radial reconstruction. Although a small systematic bias was detected in the radial data, both methods demonstrated a strong correlation and comparable clinical interpretability in evaluating pancreatic steatosis (Figure 7). The residual bias may be partially attributed to lingering motion artifacts, variations in the water‐fat separation algorithms used for radial and Cartesian data, and differences in the applied imaging parameters—particularly the echo times.

The present work tests the free‐breathing radial technique in the scenario of a LION study in people with obesity. For both the radial and the Cartesian scan, a statistically significant reduction in mean pancreatic PDFF was observed at the follow‐up scan compared with the baseline, indicating that weight loss is associated with mean pancreatic PDFF reduction (Figure 8). Moreover, a statistically significant reduction of the pancreatic volume was found. Hence, both sequences provide comparable clinical value in the evaluation of pancreatic steatosis over the duration of the LION study setting. The reduction of pancreatic fat content and volume during weight loss has already been reported in the literature. In a study cohort of 2021 subjects, Saisho et al. [6] used computed tomography and established that the total (~32%), parenchymal (~13%), and fat content (~68%) were all increased in subjects suffering from obesity. Rupp et al. [22] used a different and larger subset of the LION MRI dataset; however, for this dataset, they also confirmed the pancreatic mean PDFF reduction after the weight loss intervention. There, a mean pancreatic PDFF change from 15.0 [interquartile range: 10.9–23.0] % at baseline to 8.2 [interquartile range: 7.1–11.4] % during a follow‐up visit 1 year later was reported. Jiang et al. [51] have also observed a reduction in pancreatic fat fraction following a diet‐induced weight loss using MRI. In a study cohort of 137 nondiabetic obese adults, a significant decrease in pancreatic fat content was measured when the weight loss exceeded 7.5%. However, the reduction in pancreatic fat content was reported to be dependent on the loss of visceral adipose tissue volume.

The proposed method presents some limitations. First, the quality of the free‐breathing self‐navigated radial reconstruction relies heavily on the breathing curve of the scanned subject, which requires a sufficient sampling of the chosen reconstructed motion state (end‐expiration). While in this dataset no significant outliers were reported, incomplete expirations during the scan sequence can significantly affect the efficacy of the self‐navigation. In these cases, the reconstruction of a different motion state might be necessary. Second, while the radial SoS trajectory allows for a free‐breathing acquisition ensuring maximized patient comfort and cooperation, it also extends the scan time substantially to almost 3 min. In clinical settings where time is limited, the introduction of such a longer sequence might be difficult. Moreover, due to the need for an opposite spoke for the calculation of the linear phase offset due to eddy currents, in‐plane undersampling is not easily possible for this sequence. However, this can be mitigated by the use of other eddy current corrections such as RING, which only requires a few radial intersections to describe the individual spoke shifts [52]. The use of dedicated reference lines for eddy current correction at the beginning of the scan can also be considered but might suffer from nonsteady state conditions and a respiratory motion state bias. Instead of employing free‐breathing sequences such as the radial SoS, recent work by Rupp et al., also using the LION dataset, suggested addressing the long BH durations by introducing a higher acceleration factor [22]. This effectively reduced the BH duration from 16.9 to 10.3 s, at the expense of increased noise in the PDFF maps. Third, a broader validation in a larger patient cohort remains necessary. Due to large volumetric and morphological changes of the pancreas depending on the age of the person, a more differentiated analysis for different age groups is required to study the quantification accuracy and precision of the proposed free‐breathing radial SoS sequence. Finally, the literature is still contradictory regarding the implications of pancreatic fat for metabolic diseases [4, 5, 6, 7, 8, 10, 11, 12, 13, 14]. Discrepancies can possibly be attributed to widely varying image analysis methodologies, which make a consistent correlation between findings difficult. Further work is needed to investigate the exact relationship between changes in pancreatic fat and metabolic health markers.

## 5 Conclusion

This work shows that a six‐echo, free‐breathing, high‐resolution radial SoS acquisition robustly quantifies pancreatic fat at high spatial resolution and provides similar quantification results to a clinical Cartesian six‐echo BH scan. Reliable pancreatic segmentations can be obtained from whole‐body segmentation networks such as the VibeSegmentator only when manually reducing the effect of included visceral fat in the segmentation. Both the radial and Cartesian sequences showed a reduction of mean pancreatic PDFF and volume during a weight loss intervention study in individuals with obesity.

## Author Contributions

M.W., A.R., H.H., C.H., D.J., and D.C.K. designed and performed the study. R.G. and J.K. provided the framework for the VibeSegmentator. P.B. and J.S. worked on the reconstruction pipeline. P.B., C.Z., and M.W. analyzed and interpreted the data. P.B. and D.C.K. wrote the manuscript. All authors read, revised, and approved the final version and made substantial contributions to its content.

## Funding

This study has received support by the German Federal Ministry of Education and Research (BMBF, grant number 01EA1709) within the framework of the Junior Research Group “Personalized Nutrition & eHealth (PeNut)” of the enable Nutrition Cluster and by the German Research Foundation (project number 450799851 and project number 455422993/FOR5298‐iMAGO‐P1).

## Conflicts of Interest

J.K. is a shareholder of Bonescreen GmbH (Munich, Germany); J.K. received speaker honoraria from Novartis, Bracco, and Philips. H.H. is a member of the scientific advisory board of Oviva AG (Zurich, Switzerland), C.H. is a member of 4sigma GmbH (Oberhaching, Germany). H.H. and C.H. received speaker honoraria from Novo Nordisk (Copenhagen, Denmark), C.H. received speaker honoraria from Eli Lilly and Company. C.H. and H.H. had a research cooperation with Oviva AG (Potsdam, Germany) and Una Health GmbH (Hamburg, Germany). D.K. received grant support from Philips Healthcare while at the Technical University of Munich.

## Supporting information


**Figure S1:** T2‐weighted TSE image (left) and PDFF image obtained from the Cartesian BH scan with the overlaid manually refined segmentation image (right). The segmented pancreatic outline is visualized in bright green. Both images use the same acquired voxel size of 2 × 2 × 3 mm^3^.
**FIGURE S2:** In‐phase images from the Cartesian and radial scans of a large water phantom (300 × 300 × 100 mm^3^), used for SNR calculation via the two‐image subtraction method. Each scan was acquired twice, with an approximately 5‐min interval between acquisitions. The red dashed rectangle indicates the ROI used for the SNR calculation, encompassing approximately 75% of the total phantom volume. To simulate the undersampling inherent to self‐navigation, the radial scan was also reconstructed using the motion curve of a representative volunteer, utilizing only the data in the first motion state. The resulting SNRs are as follows: 88 for then Cartesian; 79 for the fully sampled radial; 63 for the radial using only data from the first motion state. Slight streaking artifacts are visible in the radial image reconstructed from a single bottom motion state (green arrow).

## Data Availability

The data that support the findings of this study are available on reasonable request to the corresponding author. The data are not publicly available due to privacy or ethical restrictions.

## References

[1] M. Hori , M. Takahashi , N. Hiraoka , et al., “Association of Pancreatic Fatty Infiltration With Pancreatic Ductal Adenocarcinoma,” Clinical and Translational Gastroenterology 5, no. 3 (2014): e53, 10.1038/ctg.2014.5. PMC3972693 24622469

[2] M. Takahashi , M. Hori , R. Ishigamori , M. Mutoh , T. Imai , and H. Nakagama , “Fatty Pancreas: A Possible Risk Factor for Pancreatic Cancer in Animals and Humans,” Cancer Science 109, no. 10 (2018): 3013–3023, 10.1111/cas.13766. PMC6172058 30099827

[3] Y. Suminaga , K. Namai , M. Ota , F. Konishi , N. Toyama , and H. Kamiyama , “Pancreas Head Carcinoma With Total Fat Replacement of the Dorsal Exocrine Pancreas,” Journal of Gastroenterology 39, no. 1 (2004): 76–80, 10.1007/s00535-003-1250-4. 14767740

[4] M. N. Walters , “Adipose Atrophy of the Exocrine Pancreas,” Journal of Pathology and Bacteriology 92, no. 2 (1966): 547–557, 10.1002/path.1700920232. 5964381

[5] E. Rosso , S. Casnedi , P. Pessaux , et al., “The Role of “Fatty Pancreas” and of BMI in the Occurrence of Pancreatic Fistula After Pancreaticoduodenectomy,” Journal of Gastrointestinal Surgery 13, no. 10 (2009): 1845–1851, 10.1007/s11605-009-0974-8. 19639369

[6] Y. Saisho , A. E. Butler , J. J. Meier , et al., “Pancreas Volumes in Humans From Birth to Age One Hundred Taking Into Account Sex, Obesity, and Presence of Type‐2 Diabetes,” Clinical Anatomy 20, no. 8 (2007): 933–942, 10.1002/ca.20543. PMC2680737 17879305

[7] J. S. Lee , S. H. Kim , D. W. Jun , et al., “Clinical Implications of Fatty Pancreas: Correlations Between Fatty Pancreas and Metabolic Syndrome,” World Journal of Gastroenterology 15, no. 15 (2009): 1869–1875, 10.3748/wjg.15.1869. PMC2670415 19370785

[8] B. H. Stamm , “Incidence and Diagnostic Significance of Minor Pathologic Changes in the Adult Pancreas at Autopsy,” Human Pathology 15, no. 7 (1984): 677–683, 10.1016/S0046-8177(84)80294-4. 6745910

[9] A. Heuck , P. A. Maubach , M. Reiser , et al., “Age‐Related Morphology of the Normal Pancreas on Computed Tomography,” Gastrointestinal Radiology 12, no. 1 (1987): 18–22, 10.1007/BF01885094. 3792751

[10] J. Xie , L. Xu , Y. Pan , et al., “Nonalcoholic Fatty Pancreas Disease Is Related Independently to the Severity of Acute Pancreatitis,” European Journal of Gastroenterology & Hepatology 31, no. 8 (2019): 973–978, 10.1097/MEG.0000000000001477. 31233410

[11] D. M. Tremmel , A. K. Feeney , S. A. Mitchell , et al., “Hypertension, but Not Body Mass Index, Is Predictive of Increased Pancreatic Lipid Content and Islet Dysfunction,” American Journal of Transplantation 20, no. 4 (2020): 1105–1115, 10.1111/ajt.15698. PMC7103563 31715064

[12] K. A. Lê , E. E. Ventura , J. Q. Fisher , et al., “Ethnic Differences in Pancreatic Fat Accumulation and Its Relationship With Other Fat Depots and Inflammatory Markers,” Diabetes Care 34, no. 2 (2011): 485–490, 10.2337/dc10-0760. PMC3024373 21270204

[13] K. Panc , H. Gundogdu , S. Sekmen , M. Basaran , and E. Gurun , “Liver and Pancreatic Fat Fractions as Predictors of Disease Severity in Acute Pancreatitis: An MRI IDEAL‐IQ Study,” Abdominal Radiology 50, no. 8 (2025): 3734–3743, 10.1007/s00261-025-04809-y. PMC12267334 39883165

[14] A. T. Trout , D. E. Hunte , M. Mouzaki , et al., “Relationship Between Abdominal Fat Stores and Liver Fat, Pancreatic Fat, and Metabolic Comorbidities in a Pediatric Population With Non‐Alcoholic Fatty Liver Disease,” Abdominal Radiology 44, no. 9 (2019): 3107–3114, 10.1007/s00261-019-02123-y. 31312893

[15] W. T. Dixon , “Simple Proton Spectroscopic Imaging,” Radiology 153, no. 1 (1984): 189–194, 10.1148/radiology.153.1.6089263. 6089263

[16] M. Bydder , T. Yokoo , G. Hamilton , et al., “Relaxation Effects in the Quantification of Fat Using Gradient Echo Imaging,” Magnetic Resonance Imaging 26, no. 3 (2008): 347–359, 10.1016/j.mri.2007.08.012. PMC2386876 18093781

[17] H. Yu , A. Shimakawa , C. A. McKenzie , E. Brodsky , J. H. Brittain , and S. B. Reeder , “Multiecho Water‐Fat Separation and Simultaneous R2* Estimation With Multifrequency Fat Spectrum Modeling,” Magnetic Resonance in Medicine 60, no. 5 (2008): 1122–1134, 10.1002/mrm.21737. PMC3070175 18956464

[18] S. B. Reeder and C. B. Sirlin , “Quantification of Liver Fat With Magnetic Resonance Imaging,” Magnetic Resonance Imaging Clinics of North America 18, no. 3 (2010): 337–357, 10.1016/j.mric.2010.08.013. PMC3002753 21094444

[19] J. Starekova , D. Hernando , P. J. Pickhardt , and S. B. Reeder , “Quantification of Liver Fat Content With CT and MRI: State of the Art,” Radiology 301, no. 2 (2021): 250–262, 10.1148/radiol.2021204288. PMC8574059 34546125

[20] S. B. Reeder , P. M. Robson , H. Yu , et al., “Quantification of Hepatic Steatosis With MRI: The Effects of Accurate Fat Spectral Modeling,” Journal of Magnetic Resonance Imaging 29, no. 6 (2009): 1332–1339, 10.1002/jmri.21751. PMC2689318 19472390

[21] P. S. Dulai , C. B. Sirlin , and R. Loomba , “MRI and MRE for Non‐Invasive Quantitative Assessment of Hepatic Steatosis and Fibrosis in NAFLD and NASH: Clinical Trials to Clinical Practice,” Journal of Hepatology 65, no. 5 (2016): 1006–1016, 10.1016/j.jhep.2016.06.005. PMC5124376 27312947

[22] S. Rupp , J. Han , S. M. Naebauer , et al., “Acceleration of Chemical Shift Encoding‐Based Water‐Fat Imaging for Pancreatic Proton Density Fat Fraction Mapping in a Single Breath‐Hold: Data From the LION Study,” European Journal of Radiology 195 (2026): 112641, 10.1016/j.ejrad.2025.112641. 41499911

[23] M. D. Chouhan , L. Firmin , S. Read , Z. Amin , and S. A. Taylor , “Quantitative Pancreatic MRI: A Pathology‐Based Review,” British Journal of Radiology 92, no. 1099 (1099): 20180941, 10.1259/bjr.20180941. PMC6636277 30982337

[24] H. Zhao , Q. Xu , R. Gao , et al., “Clinical Feasibility of 5.0 T MRI/MRCP in Characterizing Pancreatic Cystic Lesions: Comparison With 3.0 T and MDCT,” Diagnostics 14, no. 21 (2024): 2457, 10.3390/diagnostics14212457. PMC11545645 39518424

[25] R. Ehman , M. McNamara , M. Pallack , H. Hricak , and C. Higgins , “Magnetic Resonance Imaging With Respiratory Gating: Techniques and Advantages,” American Journal of Roentgenology 143, no. 6 (1984): 1175–1182, 10.2214/ajr.143.6.1175. 6333787

[26] C. Arboleda , D. Aguirre‐Reyes , M. P. García , et al., “Total Liver Fat Quantification Using Three‐Dimensional Respiratory Self‐Navigated MRI Sequence,” Magnetic Resonance in Medicine 76, no. 5 (2016): 1400–1409, 10.1002/mrm.26028. 26588040

[27] M. Schneider , T. Benkert , E. Solomon , et al., “Free‐Breathing Fat and R 2 * Quantification in the Liver Using a Stack‐of‐Stars Multi‐Echo Acquisition With Respiratory‐Resolved Model‐Based Reconstruction,” Magnetic Resonance in Medicine 84, no. 5 (2020): 2592–2605, 10.1002/mrm.28280. PMC7396291 32301168

[28] R. Zhao , Y. Zhang , X. Wang , et al., “Motion‐Robust, High‐SNR Liver Fat Quantification Using a 2D Sequential Acquisition With a Variable Flip Angle Approach,” Magnetic Resonance in Medicine 84, no. 4 (2020): 2004–2017, 10.1002/mrm.28263. PMC7366366 32243665

[29] B. D. Pooler , D. Hernando , J. A. Ruby , H. Ishii , A. Shimakawa , and S. B. Reeder , “Validation of a Motion‐Robust 2D Sequential Technique for Quantification of Hepatic Proton Density Fat Fraction During Free Breathing,” Journal of Magnetic Resonance Imaging 48, no. 6 (2018): 1578–1585, 10.1002/jmri.26056. PMC6589341 29665193

[30] P. Braun , J. Stelter , S. Ruschke , et al., “Free‐Breathing Fat Quantification Using a Phase Error‐Corrected Cartesian Acquisition With Spiral Profile Ordering,” Magnetic Resonance in Medicine 96 (2026): 1709–1725, 10.1002/mrm.70474. PMC13418965 42304796

[31] T. Armstrong , I. Dregely , A. Stemmer , et al., “Free‐Breathing Liver Fat Quantification Using a Multiecho 3D Stack‐of‐Radial Technique,” Magnetic Resonance in Medicine 79, no. 1 (2018): 370–382, 10.1002/mrm.26693. PMC5837283 28419582

[32] J. D. Story , S. Ghahremani , S. G. Kafali , et al., “Using Free‐Breathing mri to Quantify Pancreatic Fat and Investigate Spatial Heterogeneity in Children,” Journal of Magnetic Resonance Imaging 57, no. 2 (2023): 508–518, 10.1002/jmri.28337. PMC9805469 35778376

[33] A. Reik and C. Holzapfel , “Randomized Controlled Lifestyle Intervention (LION) Study for Weight Loss and Maintenance in Adults With Obesity—Design and Methods,” Frontiers in Nutrition 7 (2020): 586985, 10.3389/fnut.2020.586985. PMC7683381 33240920

[34] B. T. Svedin , A. Payne , B. D. Bolster , and D. L. Parker , “Multiecho Pseudo‐Golden Angle Stack of Stars Thermometry With High Spatial and Temporal Resolution Using k‐Space Weighted Image Contrast,” Magnetic Resonance in Medicine 79, no. 3 (2018): 1407–1419, 10.1002/mrm.26797. PMC6711157 28643383

[35] H. Eggers , B. Brendel , A. Duijndam , and G. Herigault , “Dual‐Echo Dixon Imaging With Flexible Choice of Echo Times,” Magnetic Resonance in Medicine 65, no. 1 (2011): 96–107, 10.1002/mrm.22578. 20860006

[36] S. Ruschke , H. Eggers , H. Kooijman , et al., “Correction of Phase Errors in Quantitative Water–Fat Imaging Using a Monopolar Time‐Interleaved Multi‐Echo Gradient Echo Sequence,” Magnetic Resonance in Medicine 78, no. 3 (2017): 984–996, 10.1002/mrm.26485. 27797100

[37] S. Rosenzweig , N. Scholand , H. C. M. Holme , and M. Uecker , “Cardiac and Respiratory Self‐Gating in Radial MRI Using an Adapted Singular Spectrum Analysis (SSA‐FARY),” IEEE Transactions on Medical Imaging 39, no. 10 (2020): 3029–3041, 10.1109/TMI.2020.2985994. 32275585

[38] C. Boehm , M. N. Diefenbach , M. R. Makowski , and D. C. Karampinos , “Improved Body Quantitative Susceptibility Mapping by Using a Variable‐Layer Single‐Min‐Cut Graph‐Cut for Field‐Mapping,” Magnetic Resonance in Medicine 85, no. 3 (2021): 1697–1712, 10.1002/mrm.28515. 33151604

[39] J. Berglund and J. Kullberg , “Three‐Dimensional Water/Fat Separation and T Estimation Based on Whole‐Image Optimization—Application in Breathhold Liver Imaging at 1.5 T,” Magnetic Resonance in Medicine 67, no. 6 (2012): 1684–1693, 10.1002/mrm.23185. 22189760

[40] B. W. Murphy , P. L. Carson , J. H. Ellis , Y. T. Zhang , R. J. Hyde , and T. L. Chenevert , “Signal‐to‐Noise Measures for Magnetic Resonance Imagers,” Magnetic Resonance Imaging 11, no. 3 (1993): 425–428, 10.1016/0730-725X(93)90076-P. 8505876

[41] F. L. Goerner and G. D. Clarke , “Measuring Signal‐to‐Noise Ratio in Partially Parallel Imaging MRI,” Medical Physics 38, no. 9 (2011): 5049–5057, 10.1118/1.3618730. PMC3170395 21978049

[42] National Electrical Manufacturers Association , “Determination of Signal‐to‐Noise Ratio (SNR) in Diagnostic Magnetic Resonance Imaging,” (2008).

[43] R. Graf , P. Platzek , E. O. Riedel , et al., “VIBESegmentator: Full Body MRI Segmentation for the NAKO and UK Biobank,” European Radiology 36 (2025): 2548–2562, 10.1007/s00330-025-12035-9. PMC13035649 41068435

[44] P. A. Yushkevich , J. Piven , H. C. Hazlett , et al., “User‐Guided 3D Active Contour Segmentation of Anatomical Structures: Significantly Improved Efficiency and Reliability,” NeuroImage 31, no. 3 (2006): 1116–1128, 10.1016/j.neuroimage.2006.01.015. 16545965

[45] J. Virostko , R. C. Craddock , J. M. Williams , et al., “Development of a Standardized MRI Protocol for Pancreas Assessment in Humans. Jin M, ed,” PLoS ONE 16, no. 8 (2021): e0256029, 10.1371/journal.pone.0256029. PMC8384163 34428220

[46] A. Al‐Mrabeh , K. G. Hollingsworth , S. Steven , D. Tiniakos , and R. Taylor , “Quantification of Intrapancreatic Fat in Type 2 Diabetes by MRI,” PLoS ONE 12, no. 4 (2017): e0174660, 10.1371/journal.pone.0174660. PMC5378354 28369092

[47] W. Zhang , S. G. Kafali , T. Adamos , et al., “Cross‐Cohort Federated Learning for Pediatric Abdominal Adipose Tissue Segmentation and Quantification Using Free‐Breathing 3D MRI,” Radiology Advances 3, no. 1 (2026): umag002, 10.1093/radadv/umag002. PMC12916172 41717043

[48] X. Zhong , H. H. Hu , T. Armstrong , et al., “Free‐Breathing Volumetric Liver and Proton Density Fat Fraction Quantification in Pediatric Patients Using Stack‐of‐Radial MRI With Self‐Gating Motion Compensation,” Journal of Magnetic Resonance Imaging 53, no. 1 (2021): 118–129, 10.1002/jmri.27205. 32478915

[49] S. Kato , A. Iwasaki , Y. Kurita , et al., “Three‐Dimensional Analysis of Pancreatic Fat by Fat‐Water Magnetic Resonance Imaging Provides Detailed Characterization of Pancreatic Steatosis With Improved Reproducibility. Bray TJ, ed,” PLoS ONE 14, no. 12 (2019): e0224921, 10.1371/journal.pone.0224921. PMC6886808 31790429

[50] S. Matsumoto , H. Mori , H. Miyake , et al., “Uneven Fatty Replacement of the Pancreas: Evaluation With CT,” Radiology 194, no. 2 (1995): 453–458, 10.1148/radiology.194.2.7824726. 7824726

[51] Y. Jiang , M. Spurny , R. Schübel , et al., “Changes in Pancreatic Fat Content Following Diet‐Induced Weight Loss,” Nutrients 11, no. 4 (2019): 912, 10.3390/nu11040912. PMC6521168 31018616

[52] S. Rosenzweig , H. C. M. Holme , and M. Uecker , “Simple Auto‐Calibrated Gradient Delay Estimation From Few Spokes Using Radial Intersections (RING),” Magnetic Resonance in Medicine 81, no. 3 (2019): 1898–1906, 10.1002/mrm.27506. 30489652

