# Supplementary material for: Quantifying Pancreatic Fat Using a Free‐Breathing Acquisition in Adults With Obesity Undergoing Diet‐Induced Weight Loss—Results From the LION Study
Source: NMR Biomed. 2026 Sep 25;39(11):e70407. doi: 10.1002/nbm.70407 (PMC13613249; doi:10.1002/nbm.70407)
Supplement: Supplementary file 1 [file NBM-39-e70407-s001.docx]

**Quantifying pancreatic fat using a free-breathing acquisition in adults with obesity undergoing diet-induced weight loss — results from the LION study**

**Supplementary material**

FIG. S1: T2-weighted TSE image (left) and PDFF image obtained from the Cartesian BH scan with the overlaid manually refined segmentation image (right). The segmented pancreatic outline is visualized in bright green. Both images use the same acquired voxel size of 2 x 2 x 3 mm^3^.

FIG. S2: In-phase images from the Cartesian and radial scans of a large water phantom (300 x 300 x 100 mm^3^), used for SNR calculation via the two-image subtraction method. Each scan was acquired twice, with an approximately 5-minute interval between acquisitions. The red dashed rectangle indicates the ROI used for the SNR calculation, encompassing approximately 75% of the total phantom volume. To simulate the undersampling inherent to self-navigation, the radial scan was also reconstructed using the motion curve of a representative volunteer, utilizing only the data in the first motion state. The resulting SNRs are as follows: 88 for then Cartesian; 79 for the fully sampled radial; 63 for the radial using only data from the first motion state. Slight streaking artifacts are visible in the radial image reconstructed from a single bottom motion state (green arrow).
